# Multisystem inflammatory syndrome in Indian adolescents associated with SARS-CoV-2 infection: a case report

**DOI:** 10.1186/s43162-021-00085-6

**Published:** 2021-12-06

**Authors:** Rahul D. Bhiwgade, M. C. Nischitha, Bhushan Shahare, Shobhna Bitey

**Affiliations:** Department of Medicine, Indira Gandhi Government Medical, College & Hospital, Central Ave., Mominpura, Nagpur, Maharashtra 440018 India

**Keywords:** Adolescents, COVID-19, Inflammatory markers, Multisystem inflammatory syndrome, SARS-CoV-2, Shock

## Abstract

**Background:**

Adolescents with coronavirus disease 2019 (COVID-19) associated multisystem inflammatory syndrome (MIS) can present with shock and myocardial injury and mimic Kawasaki disease.

**Case presentation:**

We describe 4 previously well adolescents (age 13–14 years), presenting with clinical features of MIS in children (MIS-C). All patients had nearly similar clinical presentation. Hematological investigations revealed elevated inflammatory markers, anemia, thrombocytopenia, and decreased neutrophil:lymphocyte ratio. All patients were negative on real-time polymerase chain reaction against severe acute respiratory syndrome coronavirus 2, but had elevated immunoglobulin G titers. Two patients had atypical Kawasaki disease. Three patients had severe disease with hypotensive shock and required intensive care with fluids and inotropes. Two patients required non-invasive respiratory support for dyspnea and one patient had biventricular dysfunction. All received empiric antibiotics, low-molecular weight heparin, steroids, and intravenous immunoglobulin. One patient succumbed, while others recovered well.

**Conclusions:**

MIS-C may be a late presentation in adolescent with COVID-19. Individualized treatment with empiric antibiotics, immunomodulation, and thromboprophylaxis can result in significantly better outcome.

## Background

The coronavirus disease 2019 (COVID-19) pandemic has affected all the countries and age-groups alike. However, during initial part of pandemic, COVID-19 affected children with milder form of disease and had better clinical outcomes than adults [1, 2]. Subsequently, a rising number of previously well children with severe acute respiratory syndrome coronavirus-2 (SARS-CoV-2) induced hyperinflammatory states resembling macrophage activation syndrome or hemophagocytic lymphohistiocytosis, toxic shock syndrome (TSS), and Kawasaki disease (KD) were reported [3–5]. The Centers for Disease Control and Prevention termed this condition as multi-system inflammatory syndrome in children (MIS-C) [6].

Here, we describe 4 adolescents with COVID-19-associated MIS-C presenting to a tertiary care center in Central India between 17 May and 17 June 2021. They had distinct clinical features, but similar laboratory and radiological findings. Though, none of them were positive for SARS-CoV-2 nucleic acid on real-time polymerase chain reaction (RT-PCR), all of them had elevated immunoglobulin G (IgG) titers against SARS-CoV-2.

## Case presentation

**Fig. 1:**
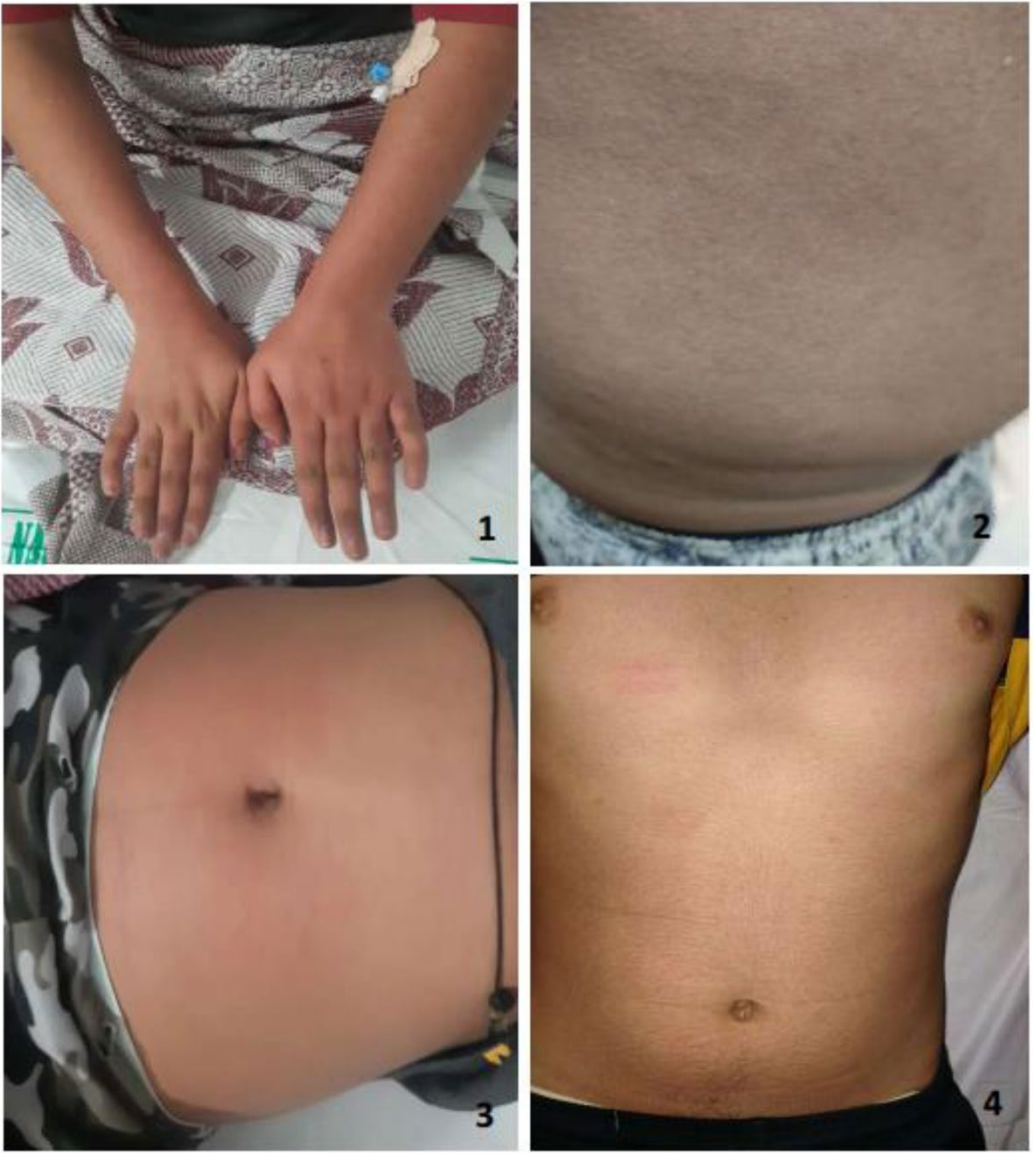
Distribution of rash in patients 1–4

**Fig. 2:**
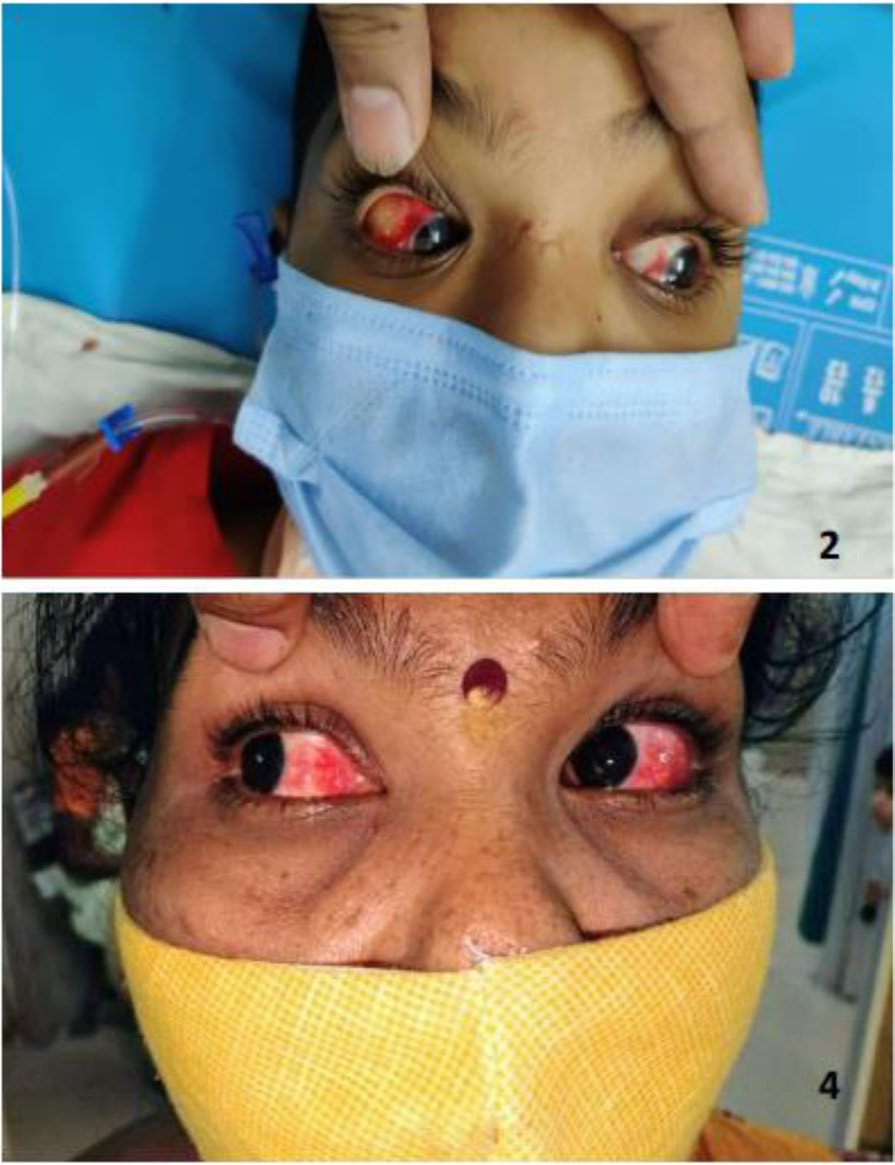
Conjunctival congestion in patients 2 and 4

Four previously well adolescents, aged 13–14 years, including equal number of males and females, presented with the disease onset over the past 5–10 days. They had a variety of presenting symptoms, of which fever with rash were common. Though the rash involved overall abdomen in every patient, entire upper limb and hands were additionally affected in patient 1 and 2, respectively (Fig. 1). Patients 2 and 4 had conjunctival congestion (Fig. 2). None of the patients had comorbidities, except patient 2, who was a known case of type 1 diabetes mellitus and was receiving Huminsulin. Two months back, patient 1 had a history of contact with COVID-19 positive mother, while other patients had no history of contact with COVID-19 patients. All patients had visited private clinics and received antibiotics (azithromycin, doxycycline), hydroxychloroquine, and symptomatic treatment; however, there was no relief.

At presentation, patients 2 and 3 were stable, while patients 1 and 4 were critical due to the presence of hypovolemic shock with hypotension and dyspnea. All patients, except patient 3, had tachypnea. In all patients, electrocardiography suggested sinus tachycardia and non-specific findings. Two dimensional echocardiography (2D ECHO) was normal in patients 1 and 2, while there was mild global hypokinesia with mild tricuspid and mitral regurgitation in patient 3, and biventricular dysfunction (ejection fraction: 54%) with mild pericardial effusion in patient 4. X-ray chest in all patients revealed normal lung fields. High-resolution computed tomography (HRCT) of chest in patients 1 and 2 was unremarkable.

Laboratory investigations revealed negative fever profile for malaria, dengue, scrub typhus, and leptospira in all the patients. Similarly, urine and blood culture were negative, and liver and renal function tests were within normal limits. Of 4 patients, 3 had anemia. Among them, patient 2 had moderate anemia, and patients 3 and 4 had mild anemia. Leukocytosis was present in patients 1 and 2, with neutrophilia and lymphocytosis observed in every patient. All, except patient 2, had thrombocytopenia. International normalization ratio was raised in patients 1 and 2. All patients had negative RT-PCR for SARS-CoV-2. While the levels of COVID-19 IgG antibody, C-reactive protein, D-dimer, lactate dehydrogenase, erythrocyte sedimentation rate, and procalcitonin were raised. Given the complaint of joint pain and residence of patient 1 in an area endemic to sickle cell disease (SCD), solubility test was done and this led to an incidental diagnosis of AS pattern SCD.

As patients 1 and 4 had critical disease, they were managed in medicine intensive care unit (MICU), while patient 2 and 3 were managed in wards. On day 2, patient 2 developed hypotensive shock and was shifted to MICU. The shock was managed with fluids and inotropes (Intravenous Noradrenaline (4–8 mg) infusion in 50 cc Normal Saline), and dyspnea was managed with 6–8 L O_2_ through bag-mask-ventilation, which improved arterial blood saturation. Additionally, in all the patients, MIS-C was suspected and intravenous immunoglobulin (IVIG, 2 mg/kg), Intravenous methylprednisolone (40 mg once daily), low molecular weight heparin (0.4 cc subcutaneously once daily), broad spectrum antibiotics (Intravenous Piperacillin-Tazobactam (4.5 gm thrice daily) in patient 1 and Ceftriaxone (1 gm twice daily) in all other patients), fluid therapy, and supportive care was initiated.

In patient 1, despite ongoing treatment, hypotension did not improved and patient developed cardiorespiratory arrest during intubation. Resuscitation was done but patient could not be revived back, while other patient responded well over next 48–72 h with gradual decrease in titers of inflammatory markers. In patient 4, bedside 2D ECHO suggested improved ventricular function. Patients were shifted out of MICU. Steroids were slowly tapered off and patients were discharged. The length of hospital stay was 2–10 days. Table 1 depicts the demographic and clinical characteristics with examination findings.

**Table 1:** Demographic and clinical characteristics and findings on investigations

Clinical characteristics	Patient 1	Patient 2	Patient 3	Patient 4
Age, years	13	14	14	14
Sex	Male	Male	Female	Female
History of contact	Present	Absent	Absent	Absent
Comorbidities	No	Type 1 DM	No	No
Time to presentation, days	5	6	10	7
Disease severity	Severe	Mild	Mild	Severe
Body temperature, ^o^F	102.3 (↑)	101.3 (↑)	100.4 (↑)	101.7 (↑)
Respiratory rate, per min	32 (↑)	30 (↑)	22 (N)	28 (↑)
Heart rate, per min	130 (↑)	110 (↑)	108 (↑)	118 (↑)
Blood pressure, mmHg	80/60 (↓)	100/60 (N)	90/60 (N)	70/40 (↓)
SpO_2_ on RA, %	85 (↓)	95 (N)	99 (N)	83 (↓)
COVID-19 RT-PCR	Negative	Negative	Negative	Negative
COVID-19 IgG Titer, U/ml	250 (↑)	131 (↑)	88 (↑)	72 (↑)
Hb levels, gm/dl	12.4 (N)	9.9 (↓)	10 (↓)	10.7 (↓)
Leucocyte count, cells/μl	15700 (↑)	16300 (↑)	1700 (N)	5400 (N)
Platelets, cells/μl	95000 (↓)	151000 (N)	71000 (↓)	97000 (↓)
Neutrophils, cells/μl	20.8 (↑)	80 (↑)	56 (↑)	83 (↑)
Lymphocytes, cells/μl	9.16 (↑)	16 (↑)	38 (↑)	11.3 (↑)
C-reactive protein, mg/L	110 (↑)	107.9 (↑)	84.1 (↑)	123 (↑)
ESR, mm/hr	54 (↑)	60 (↑)	47 (↑)	56 (↑)
Procalcitonin, ng/ml	0.10 (↑)	0.16 (↑)	0.08 (N)	0.12 (↑)
LDH, IU/l	352 (↑)	888 (↑)	628 (↑)	560 (↑)
D dimer, μg/l	4060 (↑)	9640 (↑)	2633 (↑)	1300 (↑)
INR	1.44 (↑)	1.21 (↑)	1.1 (N)	1.12 (N)
Fever profile	Negative	Negative	Negative	Negative
Blood culture	Negative	Negative	Negative	Negative
Urine culture	Negative	Negative	Negative	Negative
Liver function test	WNL	WNL	WNL	WNL
Renal function test	WNL	WNL	WNL	WNL
X-ray chest	WNL	WNL	WNL	WNL
HRCT chest	WNL	WNL	ND	ND
ECG	Sinus Tachycardia	Sinus Tachycardia	Sinus Tachycardia	Sinus Tachycardia
2D ECHO	WNL	WNL	Mild global hypokinesia	Biventricular dysfunction, mild pericardial effusion
Hospital stay, days	2	10	7	10

*SpO*_*2*_ *on RA* oxygen saturation on room air, *RT-PCR* real-time polymerase chain reaction, *Ab Antibody Hb* hemoglobin, *ESR* erythrocyte sedimentation rate, *LDH* lactate dehydrogenase, *INR* international normalization ratio, *Fever profile* malaria, dengue, scrub typhus, and leptospira, *WNL* within normal limits, *HRCT Chest* high-resolution computed tomography of chest, *ND* not done, *ECG* electrocardiography, *2D ECHO* two-dimensional echocardiography, ↑ increased, ↓ decreased, *N* normal, *ND* not done

## Discussion

The presentation and laboratory findings in our patients suggest that the occurrence of MIS-C may intensify in adolescents towards the later part of SARS-CoV-2 infection. The patients, in our report, highlight that SARS-CoV-2 infection may provoke a severe inflammatory syndrome despite seroconversion, when the virus may not be identified in upper respiratory tract [7]. All patients had negative RT-PCR and raised IgG titers, suggesting that they, especially patient 1, were asymptomatic for many days following the onset of disease. Similar to our report, other authors have described RT-PCR negative and SARS-CoV-2 IgG positive children with MIS-C [8, 9]. Based on these findings, we assume that a probable delayed mechanism linked to COVID-19 that may present as a late secondary hyperinflammatory syndrome.

As per the available literature, gastrointestinal symptoms and fever are predominantly reported in patients with MIS-C, and our patients had similar presenting symptoms [8, 10, 11]. Though these studies and our patients, in this report, had features of KD and TSS, only a limited number of children with MIS-C have been documented to satisfy KD diagnostic criteria and none of our patients fulfilled all criteria of classic KD. In our report, two patients had fever, rash, and conjunctival congestion and resembled the presentation of atypical KD. As per the available literature, shock, inflammation, and myocardial involvement are observed more frequently among patients with KD related to MIS-C than among patients with classic KD not related to MIS-C [10–12]. Additionally, this former group of patients have certain presenting feature of classic KD, probably because both KD and MIS-C are mediated by an unrestrained inflammatory response.

Half of the patients had normal respiratory status, while remaining half had dyspnea at presentation. However, chest radiography and HRCT chest were unremarkable. Both patients received non-invasive respiratory support and improved. Other studies have reported children with COVID-19, without MIS-C, who required MICU admission for respiratory distress [13, 14]. In a case report, of the two children with SARS-CoV-2 associated MIS-C, one developed dyspnea, but had normal chest radiography. The child responded well to non-invasive ventilation [9]. This finding highlights that patients with MIS-C can present with respiratory distress and still have normal lung fields.

MIS-C is related to shock and myocardial injury. Three of our patients had hypotensive shock. Additionally, one patient had heart failure with preserved ejection fraction. They were managed with fluids and inotropes, and the blood pressure normalized gradually. A study described 8 children with hyperinflammatory shock resembling KD or TSS. All had myocardial injury, with myocardial dysfunction, for which all received inotropic support and recovered well [8]. Another study reported 35 patients with cardiogenic shock or acute left ventricular dysfunction associated with multisystem inflammatory state. More than three quarter of them required inotropic support and improved [11]. In patients with MIS-C associated myocardial injury, with or without shock, cardiac Troponin is used as a marker [10, 12]. The patients, in this report, had hypovolemic shock and estimation of Troponin I could have led to false positive results, so it was not performed to assess myocardial injury.

Inflammatory markers were elevated in our patients, and published studies involving adults with severe COVID-19 suggest that immunomodulatory drugs have a role in improving the outcome. Increased levels of inflammatory markers are also described in other studies reporting children with MIS-C [8, 10, 11]. RECOVERY trial concluded that the use of dexamethasone in adults with severe COVID-19 requiring hospitalization, supplemental oxygen, or mechanical ventilation, results in reduced 28 days mortality [15]. Additionally, due to immunomodulatory property and better tolerability profile, human IVIG has been used as a treatment option in severe cases, and patients with MIS-C have been managed successfully with it [8, 10, 11]. We used both methylprednisolone and IVIG in our patients, and our experience was encouraging.

In adults with hyperinflammation due to severe COVID-19, coagulation disorders and thrombosis are noticed probably as a result of endothelial damage [16]. Elevated levels of D-dimer, a marker of disseminated intravascular coagulation, is associated with higher risk of mortality [16, 17]. Use of thromboprophylaxis has led to reduced risk of mortality in adults with severe COVID-19 [18]. Based on these findings and presence of high levels of D-dimer in children with MIS-C, anticoagulants have been tried in some of these children and no adverse effects are reported, although the advantage of anticoagulants in this age group remains controversial [10]. Our patients had elevated levels of D-dimer. Except patient with incidental diagnosis of SCD, all other patients received LMWH with no adverse effects, and none had thrombosis.

## Conclusions

Findings of our series suggest that COVID-19 can trigger hyperinflammatory state resulting in shock and pulmonary involvement, in some of the patients. All patients had raised inflammatory markers with biventricular dysfunction in one of the patient. The patients presented with distinct clinical features, with some mimicking atypical KD, the underlying mechanism for which still remain unclear. The physicians should be suspicious of MIS-C in adolescents presenting with fever, rash, and gastrointestinal symptoms. Personalized treatment including hemodynamic and respiratory support together with empirical antibiotics, fluids, inotropes, thromboprophylaxis, and immunomodulatory therapy was provided. Notwithstanding the requirement of MICU admission, the overall prognosis was good.

## Data Availability

Not applicable.

## Abbreviations

2D ECHO: Two-dimensional echocardiography
COVID-19: Coronavirus disease 2019
HRCT: High-resolution computed tomography
IgG: Immunoglobulin G
KD: Kawasaki disease
MICU: Medicine intensive care unit
MIS: Multisystem inflammatory syndrome
MIS-C: Multisystem inflammatory syndrome in children
RT-PCR: Real-time polymerase chain reaction
SARS-CoV-2: Severe acute respiratory syndrome coronavirus-2
SCD: Sickle cell disease
TSS: Toxic shock syndrome
